# Multiclass Posterior Probability Twin SVM for Motor Imagery EEG Classification

**DOI:** 10.1155/2015/251945

**Published:** 2015-12-22

**Authors:** Qingshan She, Yuliang Ma, Ming Meng, Zhizeng Luo

**Affiliations:** Institute of Intelligent Control and Robotics, Hangzhou Dianzi University, Hangzhou, Zhejiang 310018, China

## Abstract

Motor imagery electroencephalography is widely used in the brain-computer interface systems. Due to inherent characteristics of electroencephalography signals, accurate and real-time multiclass classification is always challenging. In order to solve this problem, a multiclass posterior probability solution for twin SVM is proposed by the ranking continuous output and pairwise coupling in this paper. First, two-class posterior probability model is constructed to approximate the posterior probability by the ranking continuous output techniques and Platt's estimating method. Secondly, a solution of multiclass probabilistic outputs for twin SVM is provided by combining every pair of class probabilities according to the method of pairwise coupling. Finally, the proposed method is compared with multiclass SVM and twin SVM via voting, and multiclass posterior probability SVM using different coupling approaches. The efficacy on the classification accuracy and time complexity of the proposed method has been demonstrated by both the UCI benchmark datasets and real world EEG data from BCI Competition IV Dataset 2a, respectively.

## 1. Introduction

Among different brain imaging techniques, electroencephalography (EEG) is commonly used due to its noninvasive acquisition, high temporal resolution, ease of use, and low cost [1]. The motor imagery based noninvasive BCI systems have provided users the ability to control movements of a computer cursor and interactive robotic wheelchairs and explore virtual environments [2]. The EEG measurement is a high dimensional scalp measurement, and thus it reflects the global cerebral electrophysiological activity. At the same time, it results in two weaknesses [3]. On the one hand, EEG is inherently multivariate and exhibits a high correlation [4] between the measured potentials at different electrodes (channels). On the other hand, the observed data are of low amplitude and sensitive to noise of biological, environmental, and instrumental origin. Due to these characteristics of EEG, adequate signal processing techniques are required to handle the problems of noise cancelation, feature extraction, and classification of multichannel EEG.

One core part of motor imagery based BCI systems is a pattern-classification process, where effective classification methods are crucial to achieving high performance. Conventional learning methods have been applied to binary classification of EEG, such as Fisher linear discriminant, *k*-nearest neighbor, artificial neural network, support vector machine (SVM) [5], Bayesian classifier [6], and hidden Markov models [7]. Owing to good generalization performance, absence of local minima, and sparse representation of solution, SVMs are combined with different feature extraction methods to improve classification performance of EEG patterns, but they do not provide posterior probability. They are actually useful for constructing a classifier producing a posterior probability instead of hard class labels, due to the fact they can be suitable for postprocessing and correcting the error introduced by wrongly labeled/noisy instances [8, 9]. Platt proposed probability output methods for SVM and validated its performance on three data-mining-style datasets [8]. Recently, Shao proposed the two-class probability estimates for twin SVM (TSVM) by making use of its superiority over SVM [10]. Different from SVMs in one fundamental way, TSVM aims to generate two nonparallel planes by solving two small size quadratic programming problems such that it is approximately four times faster than SVMs [11].

Consider the task of learning to recognize several motor imagery tasks performed by a given subject. This is a multiclass recognition problem. As for the posterior probability SVM based multiclassification, it is usually solved by using their multiclass extensions via the single-machine approach [9] and the multimachine approach [12]. The former involves directly solving a single optimization problem, while the latter involves decomposing a multiclass problem to a set of two-class problems via one-against-rest (OAR) and one-against-one (OAO). For the OAR approach, the number of data samples for a given task is a small fraction of the whole data, and thus the sizes of the two classes in each binary classification problem are unbalanced [9, 13]. By use of the OAO approach, the probability estimates for multiclass SVM have been proposed by combining all pairwise comparisons [12] and demonstrated that it is more stable than existing methods, that is, the voting scheme and the method in [14].

To the best of our knowledge, no previous work has been done for twin SVM with multiclass posterior probability outputs, or more specially multiclass posterior TSVM modeling by the ranking continuous output and pairwise coupling. The main advantage of our work is that multiclass posterior TSVM model can provide the posterior probability with high classification accuracy and low time complexity as compared with conventional SVM methods, and it is suitable for BCI applications. In order to handle multiclass classification of motor imagery EEG, a multiclass posterior probability TSVM (PPTSVM) solution is proposed by combining two-class PPTSVM with pairwise coupling in this paper. First, two-class PPTSVM model is constructed to approximate the posterior probability by the ranking continuous output techniques [10] and Platt's estimating method [8]. Secondly, a solution of multiclass probabilistic outputs for TSVM is provided by combining every pair of class probabilities according to the method of pairwise coupling [12]. Finally, the proposed method is compared with multiclass SVM via voting, multiclass TSVM by voting, and multiclass posterior probability SVM via the coupling approach. The classification accuracy and time complexity of the proposed method have been manifested by both the UCI benchmark datasets and real world EEG data from BCI Competition IV Dataset 2a.

This paper is organized as follows: Section 2 states the multiclass posterior probability TSVM model including a brief introduction to standard TSVM, two-class PPTSVM formulation and its multiclass extension via pairwise coupling.  Section 3 presents some experimental results on both benchmark datasets and motor imagery EEG datasets to demonstrate the validity of the proposed method. Finally, concluding remarks are given in Section 4.

## 2. Foundations for the Proposed Model Description

### 2.1. Formulation of Twin Support Vector Machine

In a two-class problem, the patterns to be classified are denoted by a set of *m* row vectors *A*_*i*_ ∈ *R* ^*n*^, where *A*_*i*_ = (*A*_*i*1_, *A*_*i*2_,…, *A*_*in*_), and let **y** ∈ {−1,1} represent the class to which the pattern belongs. As compared with the classical soft margin SVM classifier finding one hyperplane *w* ^*T*^ *x* + *b* = 0 that separates patterns from the two classes, TSVM seeks two nonparallel hyperplanes [11]. Let the number of patterns in classes +1 and −1 be, respectively, given by *m*_1_ and *m*_2_, where *m*_1_ + *m*_2_ = *m*. For simplicity of expression, the patterns of classes +1 and −1 are denoted by matrices *A* ∈ *R* ^*m*_1_×*n*^ and *B* ∈ *R* ^*m*_2_×*n*^, respectively. In nonlinear discrimination, the TSVM with nonlinear kernel finds the following kernel-generated surfaces:

(1) f1xKxT,CTu1+b1=0,f2xKxT,CTu2+b2=0

Here, *C* ^*T*^ = [*A* *B*]^*T*^, and *K* is an appropriately chosen kernel, where *K*(*x* ^*T*^, *C* ^*T*^) = *ϕ*(*x* ^*T*^) · *ϕ*(*C* ^*T*^) and *ϕ*(·) is a nonlinear mapping function.

The parameters of the two hypersurfaces are obtained by solving the following pair of quadratic programming problems:

(2) Minu1,b1,q 12KA,CTu1+e1b12+c1e2Tqs.t. −KB,CTu1+e2b1+q≥e2,q≥0,

(3) Minu2,b2,q 12KB,CTu2+e2b22+c2e1Tqs.t. KA,CTu2+e1b2+q≥e1,q≥0,

where the positive constants *c*_1_ and *c*_2_ are penalty parameters and *e*_1_ and *e*_2_ are the vectors of parameters of appropriate dimensions and ‖·‖ denotes the *L*_2_ norm. The objective functions have clear geometric meaning; for example, function (2) shows that the first term tends to keep the hypersurface close to points of class +1, while the constraints require the hypersurface to be at a distance of at least 1 from points of class −1.

By introducing Lagrange multipliers and exploiting Karush-Kuhn-Tucker (KKT) conditions, the Wolfe dual formulation of the above optimization problem can be obtained as follows [11]:

(4) Maxα e2Tα−12αTGHTH−1GTαs.t. 0≤α≤c1,Maxβ e1Tβ−12βTHGTG−1HTβs.t. 0≤β≤c2,

where *G* = [*K*(*B*, *C* ^*T*^) *e*_2_], *H* = [*K*(*A*, *C* ^*T*^) *e*_1_], *α* = (*α*_1_, *α*_2_,…, *α*_*m*_2__)^*T*^, and *β* = (*β*_1_, *β*_2_,…, *β*_*m*_1__)^*T*^ are the vectors of Lagrangian multipliers. Once the above dual formations are solved to obtain the surfaces (1), a new sample *x* ∈ *R* ^*n*^ is assigned to class *i* (*i* = +1, −1), depending on which of the two surfaces it lies closest to, given by

(5) $$\begin{array}{c} \text{class}\;\;i=\arg\;\underset{k=1,2}{\min}\begin{array}{c} \frac{\left|K\left(x^{T},C^{T}\right)u_{k}+b_{k}\right|}{\sqrt{u_{k}^{T}K\left(C,C^{T}\right)u_{k}}} \end{array}, \end{array}$$

where |·| is the perpendicular distance of the point *x* from the surface *K*(*x* ^*T*^, *C* ^*T*^)*u*_*k*_ + *b*_*k*_ = 0, *k* = 1,2.

### 2.2. Multiclass Posterior Probability TSVM

The multiclass extension to TSVM can also be modified to include posterior probability estimates instead of hard labels. In this section, multiclass posterior probability TSVM model is established. Firstly, similar to [10], a continuous output value is defined by calculating the distances from a sample to two decision hypersurfaces of TSVM with nonlinear kernels. Secondly, two-class posterior probability TSVM model is constructed to approximate the posterior probability using Platt's estimating method [8]. Here it is noted that the current SVM probability models cannot be used directly due to the different mechanism of TSVM and SVM. Finally, a solution of multiclass probabilistic outputs for TSVM is provided by combining every pair of class probabilities according to the pairwise coupling method [12].

#### 2.2.1. Two-Class PPTSVM Model

In TSVM binary classification, once the surfaces (1) are obtained, the label of any sample can be predicted. However, many applications require a posterior class probability instead of predicting the hard label. Since traditional TSVMs do not provide such probabilities, a modeling method of two-class PPTSVM is constructed by the following [8, 10].

To reflect the degree of new sample belonging to a certain class, let a function *f*(*x*) be the continuous output of a two-class TSVM [10]

(6) $$\begin{array}{c} f\left(\;x\;\right)=\left\{\begin{array}{cc} d_{min}\left(x\right){\left(\frac{d_{min}\left(x\right)}{d_{max}\left(x\right)}\right)}^{\gamma }, & \frac{\left|K\left(x^{T},C^{T}\right)u_{1}+b_{1}\right|}{\left|K\left(x^{T},C^{T}\right)u_{2}+b_{2}\right|}\cdot \frac{\sqrt{u_{2}^{T}K\left(C,C^{T}\right)u_{2}}}{\sqrt{u_{1}^{T}K\left(C,C^{T}\right)u_{1}}}<1,\\ 0, & \frac{\left|K\left(x^{T},C^{T}\right)u_{1}+b_{1}\right|}{\left|K\left(x^{T},C^{T}\right)u_{2}+b_{2}\right|}\cdot \frac{\sqrt{u_{2}^{T}K\left(C,C^{T}\right)u_{2}}}{\sqrt{u_{1}^{T}K\left(C,C^{T}\right)u_{1}}}=1,\\ -d_{min}\left(x\right){\left(\frac{d_{min}\left(x\right)}{d_{max}\left(x\right)}\right)}^{\gamma }, & \frac{\left|K\left(x^{T},C^{T}\right)u_{1}+b_{1}\right|}{\left|K\left(x^{T},C^{T}\right)u_{2}+b_{2}\right|}\cdot \frac{\sqrt{u_{2}^{T}K\left(C,C^{T}\right)u_{2}}}{\sqrt{u_{1}^{T}K\left(C,C^{T}\right)u_{1}}}>1, \end{array}\right. \end{array}$$

where *γ* > 0 is a weight parameter and two relevant variables *d*_min_(*x*) and *d*_max_(*x*) denote the minimum and maximum value in the distances *d*_+_(*x*) and *d*_−_(*x*) from the sample *x* to two separating hypersurfaces, respectively. Here, the distances *d*_+_(*x*) and *d*_−_(*x*) are given by

(7) d+x=KxT,CTu1+b1/u1TKC,CTu1+KxT,CTu2+b2/u2TKC,CTu22+2u1TKC,CTu2/u1TKC,CTu1u2TKC,CTu2,d−x=KxT,CTu1+b1/u1TKC,CTu1−KxT,CTu2+b2/u2TKC,CTu22−2u1TKC,CTu2/u1TKC,CTu1u2TKC,CTu2.

Here, the probability of the sample *x* belonging to class *i* (*i* = +1, −1) depends on the value of the output function *f*(*x*)∈(−*∞*, +*∞*) whose value range is similar to the continuous output in SVM. The larger the value is, the bigger the probability of the sample *x* belongs to class +1, and vice versa.

According to the posterior probability estimating method proposed by Platt [8], the posterior output of the two-class TSVM can be approximated using a parametric form of a sigmoid function which plays an important role in the classification settings,

(8) py=+1 ∣ fx=11+eafx+b,py=−1 ∣ fx=eafx+b1+eafx+b,

where *a* and *b* are the fitting parameters which can be determined using the maximum likelihood estimation. In this way, the two-class PPTSVM model is established to produce the posterior probability. Due to the fact that the posterior probability at a point is the combined effect of a number of neighboring samples, the advantage is that it can give a chance to correct the error introduced by wrongly labeled or noisy points.

#### 2.2.2. Proposed Multiclass PPTSVM Model

In multiclass pattern recognition, there are two conventional approaches to extend the binary classification problem to the multiclass one. The first method couples the constraints of having multiple classes in a single formulation. The other one aims to convert the multiclass problem to a set of independent two-class problems by different decomposition methods. Pairwise coupling is popular multiclass classification method that combines together all pairwise comparisons for each pair of classes [12]. In this section, a multiclass extension to TSVM with probabilistic outputs is proposed based on pairwise coupling due to its stability and effectiveness.

For the multiclass classification, a sample *x* needs to be discriminated to belong to one of *M* > 2 classes and the class label is denoted as *y* = {1,2,…, *M*}. The goal is to estimate the class probabilities *P*(*x*) = {*p*_*i*_(*x*)}_*i*=1_^*M*^, and the decision rule is $arg\;max_{i}\begin{array}{c} p_{i} \end{array}$ , where *p*_*i*_ = *p*(*y* = *i*∣*x*), *i* = 1,2,…, *M*. Let *r*_*ij*_ (*i* ≠ *j*) be the estimated pairwise class probabilities, and let *μ*_*ij*_ be expectation of *r*_*ij*_; that is, *μ*_*ij*_ = *E*(*r*_*ij*_) = *p*_*i*_/(*p*_*i*_ + *p*_*j*_) [12, 14]. Hastie and Tibshirani proposed the pairwise coupling approach to estimate the class probabilities by minimizing the Kullback-Leibler (KL) distance between *r*_*ij*_ and *μ*_*ij*_ [14]. Although the method outperforms the traditional voting rule wherein it only predicts a class label rather than a class probability [15], the assumptions that the sampling mode is binomial and *r*_*ij*_ are independent do not hold in the classification problem as pointed out in [14]. To solve the problem, a more stable method is proposed in [12]. The class probability vector *P*(*x*) is derived by the following optimization formulations:

(9) MinP 12∑i=1M ∑j:j≠iMrijpj−rjipi2s.t. ∑i=1Mpi=1,pi≥0.

The objective function of (9) can be replaced by

(10) $$\begin{array}{c} \underset{P}{\mathrm{Min}}\frac{1}{2}P^{T}QP,\;\text{where }Q_{ij}=\left\{\begin{array}{cc} \sum_{s:s\neq i}r_{si}^{2}, & i=j,\\ r_{ji}r_{ij}, & i\neq j, \end{array}\right. \end{array}$$

where the matrix *Q* is positive semidefinite. Its solution can be obtained by solving a linear-constrained convex quadratic programming problem [12]. The practical iterative algorithm is described as follows.

*Input*. It is the matrix *Q*. 

*Output*. It is the class probability vector *P*. 

*Process*

**Step 1 .**

Set the iteration number *t* = 0, and initialize the threshold of the iterative error eps = 0.005/*M*, the maximum number of iterations itmax = 100, and *P*_*t*_ = 1.0/*M*.

**Step 2 .**

Compute the matrix *QP*_*t*_ and *P*_*t*_^*T*^ *QP*_*t*_.

**Step 3 .**

Calculate the maximum deviation errmax = max|*QP*_*t*_ − *P*_*t*_^*T*^ *QP*_*t*_|. If errmax < eps, then the iterative process stops, and output the class probability vector *P*; otherwise go to Step 4.

**Step 4 .**

Iteratively compute *P*_*t*_ according to the following pseudocode:

 for  *t* = 1 : *M*
 { 
   Δ = (−*QP*_*t*_ + *P*_*t*_^*T*^ *QP*_*t*_)/*Q*_*tt*_
   
*P*_*t*_ = *P*_*t*_ + Δ
   
*P*_*t*_^*T*^ *QP*_*t*_ = (*P*_*t*_^*T*^ *QP*_*t*_ + Δ(Δ*Q*_*tt*_ + 2*QP*_*t*_))/(1 + Δ)^2^
   for  *i* = 1 : *M*
   { 
     
*QP*_*i*_ = (*QP*_*i*_ + Δ*Q*_*ti*_)/(1 + Δ)
     
*P*_*i*_ = *P*_*i*_/(1 + Δ)
    }
  }.

**Step 5 .**

If the iteration number is smaller than itmax, go to Step 2; otherwise stop iteration and output the class probability vector *P*.

In the implementation of multiclass PPSVM formulation, the detailed steps are given as follows:

(1) There are *M*(*M* − 1)/2 binary PPTSVM models for each possible pair of classes, and the corresponding pairwise class probabilities *r*_*ij*_ are estimated at each sample *x*.
(2) Adopt the iterative method to solve the optimization problem (10), obtaining the class probability vector $\hat{P}(x)={\left\{{\hat{p}}_{i}(x)\right\}}_{i=1}^{M}$ .
(3) The label of the test sample is discriminated according to the decision rule as $arg\;max_{i}\begin{array}{c} {\hat{p}}_{i} \end{array}$ , *i* = 1,2,…, *M*.

## 3. Results and Discussion

In this section, some experiments are conducted on UCI benchmark datasets and BCI competition datasets to study the performance of multiclassification approaches and verify the effectiveness of our proposed method. Six algorithms are used for multiclassification and separated into two groups. The first group produces hard class labels, consisting of conventional SVM and TSVM by voting [15]. The second group estimate class probabilities, including posterior probability SVM via minimizing the KL distance [14] (PPSVM_HT), posterior probability SVM by pairwise coupling [12] (PPSVM), and the proposed posterior probability TSVM via minimizing the KL distance [14] (PPTSVM_HT), as well as pairwise coupling [12] (PPTSVM).

Multiclass posterior probability TSVM is constructed based on the binary TSVM classification code (http://www.optimal-group.org/Resource/TWSVM.html) provided by Shao et al. [10] and multiclass posterior probability SVM code can refer to the mcpIncSVM package (http://www-ti.informatik.uni-tuebingen.de/~spueler/mcpIncSVM/). For all the six methods, Gaussian kernel function is chosen due to its validity and stability in experiments. All the methods are implemented in MATLAB 2013a environment on a PC with a 2.5 GHz processor and 4.0 GB RAM.

### 3.1. Experiments on UCI Datasets

First, our proposed method is evaluated on several multiclass datasets from the UCI Machine Learning Repository [16]. In this experiment, five datasets with multiple labels are chosen and the details are listed in Table 1. It is shown that the number of classes range from three to eleven, and the feature dimension is also different from two to sixteen.

In classification process, each dataset is first divided into training and testing sets with the ratio 65% : 35%. Secondly, *k*-fold cross-validation and grid search techniques are used to select the optimal kernel parameter *g* and penalty parameters *c*, *c*_1_, *c*_2_ for different methods, and then the sigmoid parameters *a*, *b* are obtained. Multiple rounds of cross-validation are performed using different partitions of training data in order to reduce variability, and the partitions are randomly generated by the “crossvalind” function in the MATLAB Bioinformatics toolbox. To ensure a fair comparison between different approaches, the same data partitions are used in cross-validation. Next, the trained models from training sets are applied to predict the testing sets. The classification process is repeated 100 times, and the average of these outcomes is the final classification rate.  Table 2 lists the best parameters for each benchmark dataset. For each dataset, there are *M*(*M* − 1)/2 binary classification models. For Zoo, a twofold cross-validation is conducted on the grid points {2^−2^,…, 2^2^} in both TSVM and SVM models because its minimum number of classes is 4. For all other four datasets, the best model parameters are searching by tenfold cross-validation from {2^−2^,…, 2^2^} in both TSVM and SVM models. The parameters *g*, *c*_1_, *c*_2_ of PPTSVM were set to be the same with TSVM, and the penalty parameters *g*, *c* of PPSVM are also set to be the same with SVM, as shown in Table 2.

**Figure 1:**
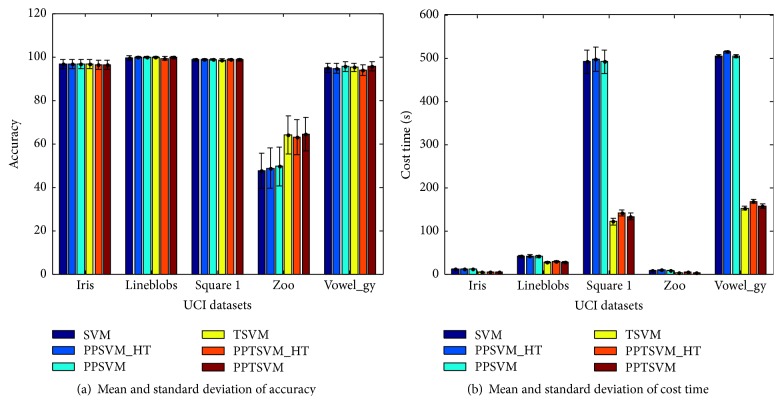
Comparisons of average and standard deviation of the classification accuracy and cost time on UCI datasets using different methods.

The performance is evaluated in terms of average value and standard deviation of the accuracy and cost time.  Figure 1 shows the classification performances for each benchmark dataset using PPTSVM, PSVM, TSVM, and SVM. It is shown that the TSVM-type classification methods perform similarly in all the five datasets, and the same is true for the SVM-type approaches. At the same time, TSVM-type competitors are consistently faster than SVM-type contenders due to the fact that TSVM-type methods solve two smaller sized quadratic programming problems instead of a single one with relatively larger size. The figure also indicates that TSVM-type classification methods yield higher accuracy in 3 of the 5 datasets (Lineblobs, Zoo, and Vowel_gy) and perform best on Zoo, while SVM-type approaches perform slightly better on two datasets (Iris and Square 1). Furthermore, PPTSVM performs better on two datasets (Zoo and Vowel_gy) than the other algorithms, especially gaining about 15% improvement over all the SVM-type contenders on the Zoo dataset. It demonstrates the potential advantage of TSVM-type model in dealing with smaller samples. However, all the classification methods obtain worse performance on the Zoo datasets because the number of its training datasets is comparatively small, and additionally it belongs to the imbalanced data in that it has seven classes with different class numbers. As proposed in [17] recently, it is interesting to construct corresponding TSVM-type models for the imbalanced classification problem although it is not the scope of this paper.

### 3.2. Applications in Classification of Motor Imagery EEG

The availability of BCI data from past competitions is an important contribution to stimulate the interdisciplinary engagement of researchers. This paper selects BCI Competition IV Dataset 2a provided by the BCI research center in Berlin, Germany [18]. Nine participants (e.g., A1~A9) were invited as experimental subjects. For each subject, four different motor imagery tasks were executed, that is, left hand, right hand, feet, and tongue. Two sessions on different days were recorded for each participant and each session was comprised of six runs separated by short breaks where one run consists of 48 trails and thus there are a total of 288 trials per session. The data were recorded using 22 Ag/AgCl electrodes. The measured signals were sampled with 250 Hz and band-pass filtered between 0.5 and 100 Hz.

Concerning the classification of motor imagery tasks, it is involved with feature extraction of EEG. The common spatial patterns (CSP) method is used to extract features since it can construct new time series whose variances are optimal for the discrimination of two populations of data [19], and thus it has been applied successfully to the classification of raw movement-related EEG. Standard CSP algorithm is proposed for binary classification, and its multiclass extensions contain using CSP within the classifier, one-versus-the-rest CSP (OVR), and simultaneous diagonalization of covariance matrices from the multiclass data [20]. The OVR approach computes the CSP features that discriminate each class from the rest of the classes [20]. CSP is extended for the multiclass data using the OVR scheme in this paper. The detailed steps of EEG processing are outlined in the following;

(1) Preprocess the multichannel EEG data using a 5-order Butterworth filter, obtaining a band-pass filtered signal with the frequency band 7–35 Hz.

(2) Perform the CSP algorithm on the filtered EEG data using the OVR scheme, getting the corresponding feature vector:

(11) $$\begin{array}{c} f_{p}=\log\left(\;\frac{\mathrm{var}\left(Z_{p}\right)}{\sum_{j=1}^{2J}\mathrm{var}\left(Z_{j}\right)}\;\right),\;p=1,\ldots ,2J, \end{array}$$

where *Z*_*p*_ denotes the signals processed by CSP, var(·) is the operation of computing vector variance, and *J* represents the number of spatial filters. For four-class data, there are 4 projection matrices, and the optimal 2*J* directions are taken in each projection matrix. When *J* = 2, the feature vector of 16 dimensions is obtained in the experiment.

(3) Recognize the classes of motor imagery tasks using the different classifiers and compare their performances evaluated in terms of mean kappa value and cost time. Used as a performance measure in BCI competition, the kappa coefficients consider the distribution of wrong classifications and the frequency of occurrence is normalized [21]. The kappa coefficient is denoted in the following equality:

(12) $$\begin{array}{c} kappa=\frac{p_{0}-p_{e}}{1-p_{e}}, \end{array}$$

where *p*_0_, the overall agreement, is equal to the classification accuracy and the chance agreement *p*_*e*_ equals the accuracy of a trivial classifier. If the actual number of samples is equally distributed across classes, the chance expected agreement is *p*_*e*_ = 1/*M*, and the kappa coefficient is given by

(13) $$\begin{array}{c} kappa=\frac{Mp_{0}-1}{M-1}. \end{array}$$

For example, an accuracy of 50% in a two-class problem is equivalent to an accuracy of 25% in a four-class problem, but the kappa value is zero in both cases, and thus it can be used to do a fair comparison of multiclass problems.

The experiment consists of two parts. In the first part, each piece of training data from BCI Competition IV Dataset 2a is randomly divided into training and testing sets with the ratio 65% : 35%, and tenfold cross-validations are performed on the cross-validation training sets, and then the trained models are applied to predict the cross-validation testing sets (part of the training data). Similar to the parameter selection process described in UCI datasets, the parameters *g*, *c*_1_, *c*_2_ of PPTSVM were set to be the same with TSVM, and the penalty parameters *g*, *c* of PPSVM are also set to be the same with SVM where the best model parameters are searching from {2^−2^,…, 2^2^} in both TSVM and SVM models.  Table 3 summarizes the best parameters for each subject in BCI Competition IV Dataset 2a.

**Figure 2:**
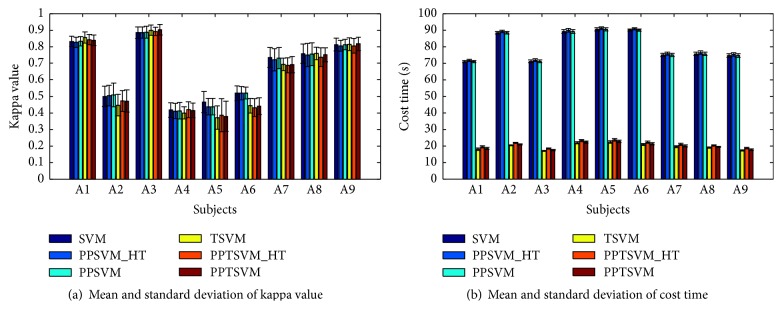
Experimental results on the mean kappa value, cost time, and their standard deviations of 10 × 10-fold cross-validation on the training data in BCI Competition IV Dataset 2a.

Figure 2 provides the classification results using kappa coefficient and cost time with mean values and standard deviations of 10 × 10-fold cross-validation. The results show that the posterior probability TSVM-type classification methods via different pairwise coupling techniques perform similarly in terms of kappa value on BCI Competition IV Dataset 2a. The same is true for different posterior probability SVM-type approaches, and the results coincide with the demonstrations in [12] when the number of classes is small; for example, it is 4 in the BCI Competition IV Dataset 2a. All the TSVM-type competitors have obvious superiority in the cost time and achieve slightly higher kappa values for 5 subjects (1, 3, 4, 8, and 9) while the SVM-type contenders perform better in 4 subjects (2, 5, 6, and 7). Over all the nine subjects, the PPTSVM method yields higher averaged mean kappa value (0.634) than TSVM (0.632) and PPTSVM_HT (0.630), but slightly lower than the best competitor SVM (0.658). Compared with the results in [22] using multiclass extensions of the CSP algorithm via the OVR approach and the naïve Bayesian Parzen window (NBPW) classifier, our method yields higher averaged mean kappa value over all the subjects than the NBPW algorithm with CSP (0.538), but lower than the NBPW algorithm with filter bank CSP approach (0.663) that employs additional feature selection based on mutual information with respect to the classical CSP algorithm. On the other hand, it is almost four times faster than the conventional SVM methods since the averaged cost time of our method is 20.040 s while that of SVM is 80.593 s, and the advantage in time complexity is helpful to BCI applications.

**Figure 3:**
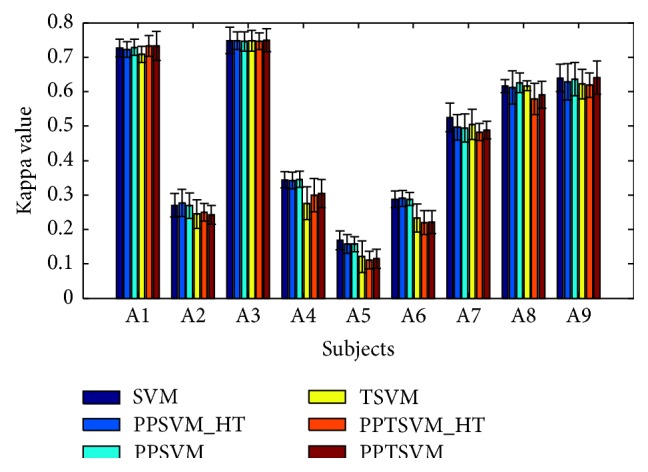
Experimental results on the mean kappa value and standard deviations of the evaluation data in BCI Competition IV Dataset 2a.

In the second part of the experiment, the trained models from cross-validation training sets are applied to the whole evaluation set. The classification results using average kappa value for all the algorithms are plotted in Figure 3. It is shown that the PPTSVM method achieves slightly higher mean kappa values in 3 subjects (1, 3, and 9). By contrast, SVM via voting yields the best averaged mean kappa value on the evaluation data (0.480) over all the nine subjects, and the PPTSVM method obtains higher averaged mean kappa value (0.454) than TSVM (0.452) and PPTSVM_HT (0.449).

Comparing the results of Figures 2 and 3, the results on the evaluation data are consistently lower than the cross-validation results for all six methods. Specially, PPTSVM yields lower mean kappa value averaged over all the subjects on the evaluation data (0.454) than the cross-validation results (0.634) in all the nine subjects.

## 4. Conclusions

In this paper, the posterior probability twin SVM is extended to the multiclass case by the ranking continuous and pairwise coupling. The performance of the proposed method has been tested in terms of classification accuracy and cost time on both the UCI benchmark datasets and real world EEG data from BCI Competition IV Datasets and compared with multiclass SVM/TSVM by the voting rule [15], multiclass posterior probability SVM/TSVM via minimizing the KL distance [14], and pairwise coupling in [12], respectively. The experimental results have demonstrated that the proposed method yields slightly higher averaged mean kappa value than TSVM by voting and PPTSVM_HT via the minimization of the KL distance on the BCI Competition IV Dataset 2a. In addition, it can achieve comparatively close performance to SVM competitors with lower time complexity on the UCI datasets and BCI Competition IV Dataset 2a. The decrease in time complexity is valuable in BCI applications and also its posterior probability output is useful for multimodal information fusion.

Although our method can yield satisfactory classification performance with low time cost, it is still designed in off-line settings, and thus it is worthwhile to research a framework for exact incremental learning and adaptation of multiclass posterior probability TSVM in future work. It is quite possible to have multiclass machine learning problem where one or more classes are rare compared with others, and constructing the TSVM-type models for the imbalanced classification problem is also part of the future work.

## Figures and Tables

**Table 1:** Descriptions of benchmark datasets used in experiments.

Datasets	Total class number	Number of each class	Feature dimension	Training samples/total samples	Testing samples/total samples
Iris	3	[50, 50, 50]	4	96/150	54/150
Lineblobs	3	[118, 75, 73]	2	173/266	93/266
Square 1	4	[250, 250, 250, 250]	2	650/1000	350/1000
Zoo	7	[41, 20, 5, 13, 4, 8, 10]	16	66/101	35/101
Vowel_gy	11	[48, 48, 48, 48, 48, 48, 48, 48, 48, 48, 48]	10	343/528	185/528

**Table 2:** Best model parameters and sigmoid parameters for five UCI datasets.

Datasets	TSVM (PPTSVM)			SVM (PPSVM)		
	*c*_1_/*c*_2_	*g*	*b*/*a*	*c*	*g*	*b*/*a*
Iris	0.25/0.25	2.25	0.4518/−8.1787	0.25	0.25	−0.0202/−4.0336
Lineblobs	0.25/0.25	0.25	0.0451/−79.2594	2.25	0.25	−0.1062/−4.8808
Square 1	0.25/0.25	3.25	−0.0967/−14.6328	1.25	1.25	0.0660/−4.9734
Zoo	0.25/0.25	0.25	0.7365/−15.2263	0.25	0.25	0.0884/−3.0649
Vowel_gy	0.25/3.25	1.25	0.2548/−93.0990	2.25	0.25	−0.2565/−4.0580

**Table 3:** Best model parameters and sigmoid parameters for each subject in BCI Competition IV Dataset 2a.

Subjects	TSVM (PPTSVM)			SVM (PPSVM)		
	*c*_1_/*c*_2_	*g*	*b*/*a*	*c*	*g*	*b*/*a*
A1	0.25/0.25	2.25	0.0021/−10.8360	1.25	3.25	0.5137/−3.1760
A2	2.25/2.25	2.25	0.1625/−143.8536	2.25	2.25	0.5102/−2.9896
A3	0.25/0.25	2.25	0.0550/−13.2102	0.25	1.25	−0.2401/−3.8991
A4	0.25/2.25	2.25	0.1680/−126.5687	1.25	1.25	−0.3945/−3.1796
A5	0.25/0.25	0.25	0.0059/−186.2766	3.25	1.25	0.6197/−3.5109
A6	0.25/2.25	1.25	0.1853/−85.6740	2.25	2.25	−0.4072/−2.6546
A7	0.25/0.25	2.25	0.0806/−21.3598	3.25	2.25	−0.1001/−2.9976
A8	0.25/1.25	1.25	0.5234/−229.3538	1.25	1.25	−0.5236/−3.7116
A9	0.25/0.25	2.25	−0.1761/−19.3798	2.25	2.25	0.3187/−3.3938
